# Computational and Spectral Investigation of 5,12-Dihydro-5,12-ethanonaphthacene-13-carbaldehyde

**DOI:** 10.3390/molecules16086741

**Published:** 2011-08-09

**Authors:** Usama Karama, Adel A. El-Azhary, Abdulrahman I. Almansour, Abdulla A. Al-Kahtani, Turki M. Al-Turki, Mohammed H. Jaafar

**Affiliations:** Chemistry Department, Faculty of Science, King Saud University, P.O. Box 2455, Riyadh 11451, Kingdom of Saudi Arabia

**Keywords:** *ab initio*, conformational analysis, IR, DFT, cycloaddition, naphthancene

## Abstract

A conformational search of 5,12-dihydro-5,12-ethanonaphthacene-13-carbaldehyde predicted the presence of twelve conformations. The geometry of the twelve conformations established at the B3LYP/6-31G* level showed only six unique ones. Vibrational frequencies were calculated at the B3LYP/6-31G* level. The calculated vibrational frequencies enabled us to interpret the appearance of two bands corresponding to the C=O stretching mode of 5,12-dihydro-5,12-ethanonaphthacene-13-carbaldehyde. The first band corresponded to the 5,12-dihydro-5,12-ethanonaphthacene-13-carbaldehyde structure where the aldehyde group O atom was above the benzene or naphthalene ring. The other band was due to the O atom of the aldehyde group pointing out of the benzene or naphthalene ring.

## 1. Introduction

Quantum chemistry, often called predictive quantum chemistry, is a useful tool for predicting structure and properties of molecules. In this manuscript the accuracy of quantum chemistry is used to understand the splitting of the C=O stretching mode of 5,12-dihydro-5,12-ethanonaphthacene-13-carbaldehyde. 

Meek *et al*. [1] have reported that the condensation of naphthacene with acrylonitrile, allyl alcohol, methyl acrylate, and acrylamide gives almost identical ratios of the possible adducts, with a slight excess of the adduct having the dienophile substituent *syn* to the benzene ring being obtained. The structures were assigned based on dipole moments. In all these adducts, there seems to be no steric or electronic factors which would influence the formation of one adduct to predominate markedly.

**Scheme 1:**
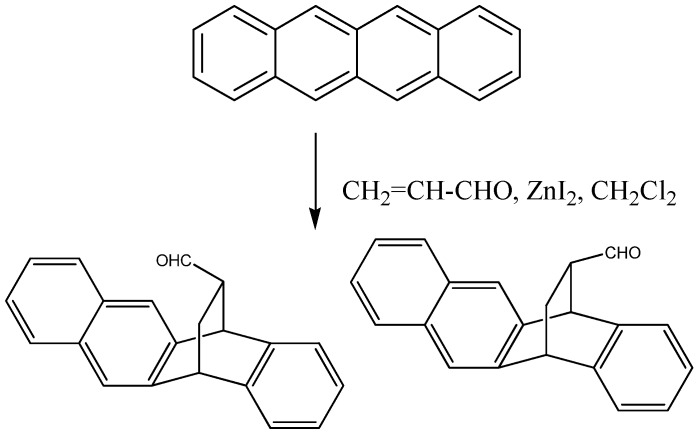
Synthesis of 5,12-dihydro-5,12-ethanonaphthacene-13-carbaldehyde**.**

In our on-going research we decided to study the outcome of the cycloaddition of naphthacene to acrolein (Scheme 1). To our surprise we obtained the possible non-separable adducts in almost a 2:1 ratio. The ratio of the isomers was determined by integration of the aldehyde peaks in ^1^H-NMR spectrum. The proton of the dominant aldehyde appeared as a doublet at 9.50 ppm and the proton of the other isomer aldehyde also appeared as a doublet at 9.48 ppm. The ethano hydrogens, bridge hydrogens and aromatic hydrogens of the two isomers showed identical shifts. The shifts of the carbon atoms in the ^13^C-NMR spectra of the two isomers showed only slight differences.

## 2. Results and Discussion

**Figure 1:**
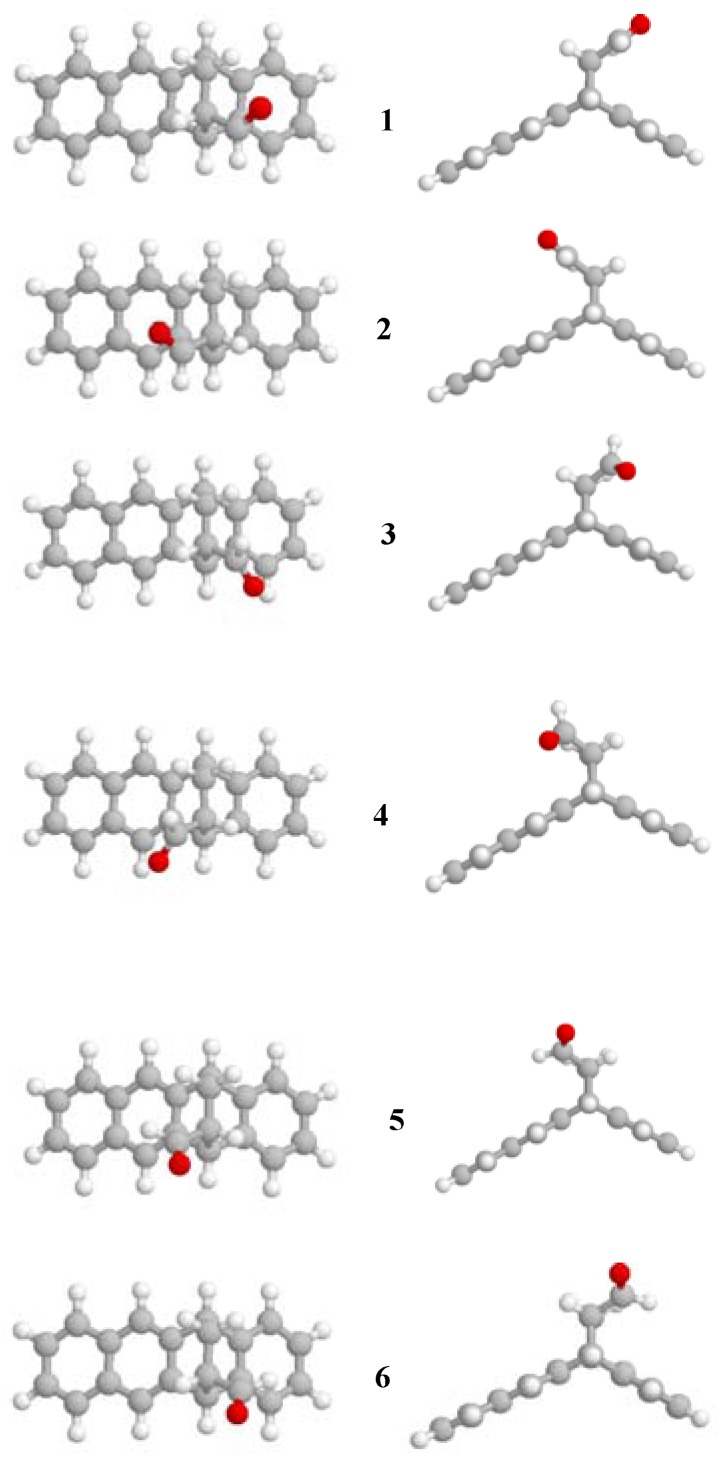
Structures of the six predicted conformations in the top and side views of 5,12-dihydro-5,12-ethanonaphthacene-13-carbaldehyde.

The structures of the six predicted conformations of 5,12-dihydro-5,12-ethanonaphthacene-13-carbaldehyde, in top and side views, are shown in Figure 1. Table 1 lists the relative energy of these six conformations with respect to the lowest energy conformation. Each of the six conformations is given a number according to MP2/6-31G*//B3LYP/6-31G* relative energy order and is indicated in boldface (Figure 1, Table 1).

It can be seen from the data in Table 1 and Figure 1 that conformation **1** with the O atom of the aldehyde group in the side of the benzene ring and above it is the lowest energy conformation. The similar conformation with the O atom of the aldehyde group in the side of the naphthalene ring and above it is the second lowest energy conformation, with an energy difference of only 0.09 kcal/mol at the MP2/6-31G*//B3LYP/6-31G* level. As will be seen shortly these two conformations are the two lowest energy conformations due to the presence of hydrogen bonds. The other four conformations have the O atom of the aldehyde group pointing out of the molecule, conformations **3** and **6** in the side of the benzene ring and conformations **4** and **5** in the side of the naphthalene ring. Conformations **5** and **6** differ from their corresponding **3** and **6** conformations in that conformation **5** and **6** have the O atom pointing upward while conformations **3** and **4** have the O atom pointing downward. Conformations **1** and **2** are stabilized by the five-membered hydrogen bond with the neighboring CH_2_ group. The O···H distance is 2.38 and 2.38 Å for conformations **1** and **2**, respectively, at the B3LYP/6-31G* level. Although it might be surprising that the O···H distance of conformations **1** and **2** are equal, but this is in fact expected considering the close resemblance of the structure and environment in both conformations.

**Table 1:** Relative energies, kcal/mol, and predicted vibrational frequency corresponding to the C=O stretching mode (cm^−1^) of the six predicted conformations of 5,12-dihydro-5,12-ethanonaphthacene-13-carbaldehyde.

Conf. No.	B3LYP/6-31G *	MP2/6-31G*//B3LYP/6-31G *	Vib. Freq.
**1**	0.00	0.00	1826
**2**	0.14	0.09	1826
**3**	0.60	0.40	1832
**4**	0.77	0.52	1832
**5**	0.83	0.96	1832
**6**	0.82	0.97	1832

**Conf. No.** is the conformation number and **Vib. Freq**. is the vibrational frequency in cm^–1^. Energy of conformation **1** at the B3LYP/6-31G* level is –885.10862 au and at the MP2/6-31G*//B3LYP/6-31G* level it is –882.29213 au.

**Figure 2:**
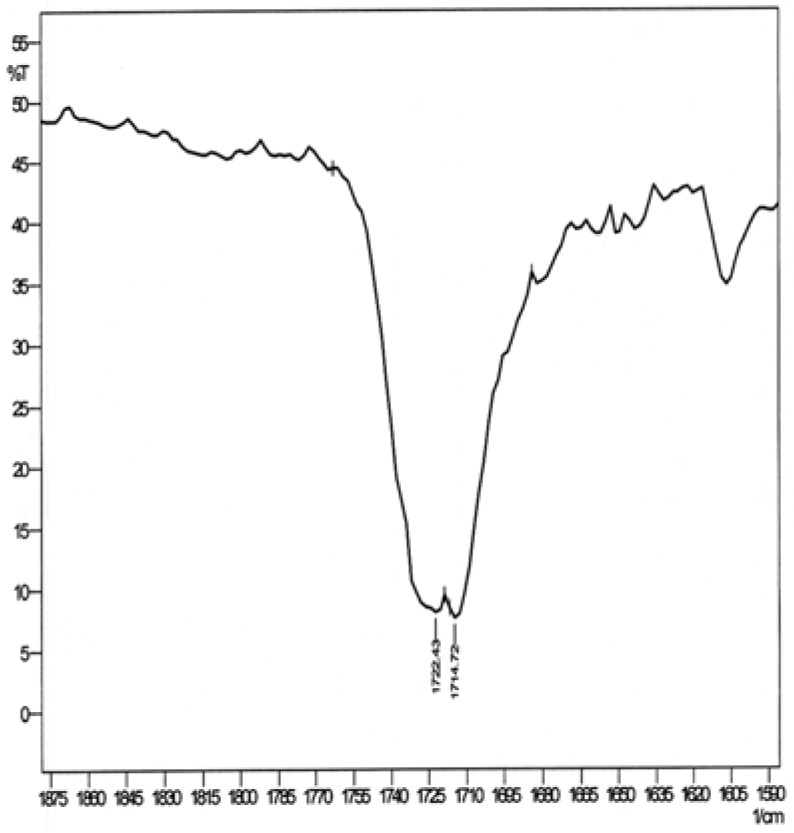
FT-IR spectrum of 5,12-dihydro-5,12-ethanonaphthacene-13-carbaldehyde.

The vibrational band corresponding to the hydrogen bonded C=O group appears at higher wave number than the non–hydrogen bonded C=O group due to the arene-carbonyl π interaction [6]. The measured IR spectrum of 5,12-dihydro-5,12-ethanonaphthacene-13-carbaldehyde is shown in Figure 2, which clearly shows that there are two bands that correspond to the C=O stretch at 1,722 and 1,714 cm^–1^. It was first thought that these two bands, which are separated by 8 cm^–1^, correspond to the two regioisomers with the aldehyde group above the naphthalene and benzene rings, respectively. The results of the conformational search in Table 1 show that, conformations **1** and **2**, which are isoenergetic and where the O atom of the aldehyde group is above the benzene or the naphthalene rings, have the same vibrational frequency corresponding to the C=O stretching mode at 1,826 cm^–1^. The other four conformations, **3**–**6**, where the O atom of the aldehyde group is either above the benzene or naphthalene rings, but pointing outside either ring have the same vibrational frequency corresponding to the C=O stretching mode at 1,832 cm^–1^.

The energy difference between these four conformations, **3**–**6**, is only 0.6 kcal/mol, according to the MP2/6-31G*//B3LYP/6-31G* level calculations. It is expected that quantum chemistry would be able to predict and interpret the vibrational C=O stretching mode of a molecule as large as 5,12-dihydro-5,12-ethanonaphthacene-13-carbaldehyde.

## 3. Experimental

The IR spectra were recorded over the range 4000-400 cm^−1 ^on a Shimadzu Prestige-21-FT-IR spectrophotometer and are expressed as ν cm^–1^. NMR spectra were recorded on a JEOL ECP 400 (400 MHz) instrument in CDCl_3_ and chemical shifts are expressed as δ ppm, and coupling constants (*J*) are given in Hertz. MS spectra and HRMS were recorded in the Department of Organic Chemistry at the University of Hannover-Germany using EI at 70 eV.

*Synthesis of 5,12-Dihydro-5,12-ethanonaphthacene-13-carbaldehyde.* A mixture of naphthacene (0.228 g, 1 mmol), acrolein (0.112 g, 2 mmol), and ZnI_2_ (0.064 g, 0.2 mmol) in 10 mL CH_2_Cl_2_ was stirred at room temperature for 24 h. The reaction mixture was concentrated and the crude product was purified by flash column chromatography on silica gel (petroleum ether-ethyl acetate 5:1) to give the product (0.21 g, 74%) as a white viscous liquid. IR (ν, neat): 3,047, 3,020, 2,929, 1,722, 1,714, 1,608, 1,500, 1,479, 1,456, 1,398, 1,076, 908, 885 cm^–1^; ^1^H-NMR (δ): 2.05-2.08 (m, 2H, H-14), 2.83-2.85 (m, 1H, H-13), 4.53 (t; *J =* 2.6Hz, 1H, H-5), 4.83 (dd; *J =* 2.5 Hz, *J =* 4.6 Hz, 1H, H-12), 7.12-7.75 (m; 10 H, aromatic-H), 9.48 (d; *J =* 1.7 Hz, H-C=O). 9.50 (d; *J =* 1.6 Hz, H-C=O); ^13^C-NMR (δ): 28.77, 28.88, 43.6426, 45.11, 45.22, 51.27, 121.74, 123.67, 124.73, 125.75, 126.34, 126.88, 127.59, 127.66, 202.47, 202.76; MS (EI) *m/z* (%): 284 (27) [M^+^], 228 (100), 226 (36), 114 (40); HRMS (EI) Calcd. for C_21_H_16_O [M^+^] 284.1201, found 284.1202.

## 4. Computations

A full conformational search was performed using the CONFLEX method fully programmed in the CONFLEX program [2,3,4]. The conformational search method was performed starting from four initial proposed conformations. These were: (1) with the O atom in the side of the benzene ring and above it; (2) with the O atom in the side of the benzene ring and pointing out of the ring; (3) the O atom in the side of the naphthalene ring above it and (4) with the O atom in the side of the naphthalene ring and pointing outside the ring. After the conformational search each of these initial conformations produced three conformations, for a total of twelve. Computations of these twelve conformations were performed at the B3LYP/6-31G* level with the correlation energy recovered at the MP2/6-31G* level. The later calculation will be referred hereafter as the MP2/6-31G*//B3LYP/6-31G* level. Only six conformations out of these twelve conformations had unique B3LYP/6-31G* energies. Vibrational frequencies were calculated for these six unique conformations at the B3LYP/6-31G* level. None of the predicted six conformations had imaginary vibrational frequencies at the B3LYP/6-31G* level. The *ab initio* calculations were performed using the G03 site of program [5].

## 5. Conclusions

It can be concluded that the appearance of two bands corresponding to the C=O stretching mode in the IR vibrational spectrum of 5,12-dihydro-5,12-ethanonaphthacene-13-carbaldehyde is due to two structures, one with the O atom of the aldehyde group above the benzene or naphthalene ring, the other is due to the O atom of the aldehyde group pointing out of the benzene or naphthalene ring.
